# Exploring female otolaryngologists’ experiences with gender bias and microaggressions: a cross sectional Canadian survey

**DOI:** 10.1186/s40463-022-00618-1

**Published:** 2023-03-06

**Authors:** Amanda C. Hu, Kelly Nguyen, Tanya K. Meyer

**Affiliations:** 1 Division of Otolaryngology-Head and Neck Surgery, University of British Columbia, 4th Floor, 2775 Laurel Street, Vancouver, BC V5Z 1M9 Canada; 2 University of British Columbia, Vancouver, Canada; 3 Department of Otolaryngology Head & Neck Surgery, University of Washington, Seattle, USA

**Keywords:** Gender bias, Otolaryngology, Microaggression, Female surgeons, Women in surgery, Self-efficacy

## Abstract

**Background:**

Gender bias is behavior that shows favoritism towards one gender over another. Microaggressions are defined as subtle, often unconscious, discriminatory, or insulting actions that communicate demeaning or negative attitudes. Our objective was to explore how female otolaryngologists experience gender bias and microaggressions in the workplace.

**Methods:**

Anonymous web-based cross-sectional Canadian survey was distributed to all female otolaryngologists (attendings and trainees) using the Dillman’s Tailored Design Method from July to August of 2021. Quantitative survey included demographic data, validated 44-item Sexist Microaggressions Experiences and Stress Scale (MESS) and validated 10-item General Self-efficacy scale (GSES). Statistical analysis included descriptive and bivariate analysis.

**Results:**

Sixty out of 200 participants (30% response rate) completed the survey (mean age 37 ± 8.3 years, 55.0% white, 41.7% trainee, 50% fellowship-trained, 50% with children, mean 9.2 ± 7.4 years of practice). Participants scored mild to moderate on the Sexist MESS—Frequency (mean ± standard deviation) 55.8 ± 24.2 (42.3% ± 18.3%), Severity 46.0 ± 23.9 (34.8% ± 18.1%), Total 104.5 ± 43.7 (39.6% ± 16.6%) and high on GSES (32.7 ± 5.7). Sexist MESS score was not associated with age, ethnicity, fellowship-training, having children, years of practice, or GSES. In the sexual objectification domain, trainees had higher frequency (p = 0.04), severity (p = 0.02) and total MESS (p = 0.02) scores than attendings.

**Conclusions:**

This was the first multicenter, Canada-wide study exploring how female otolaryngologists experience gender bias and microaggressions in the workplace. Female otolaryngologists experience mild to moderate gender bias, but have high self-efficacy to manage this issue. Trainees had more severe and frequent microaggressions than attendings in the sexual objectification domain. Future efforts should help develop strategies for all otolaryngologists to manage these experiences, and thereby improve the culture of inclusiveness and diversity in our specialty.

**Graphical Abstract:**

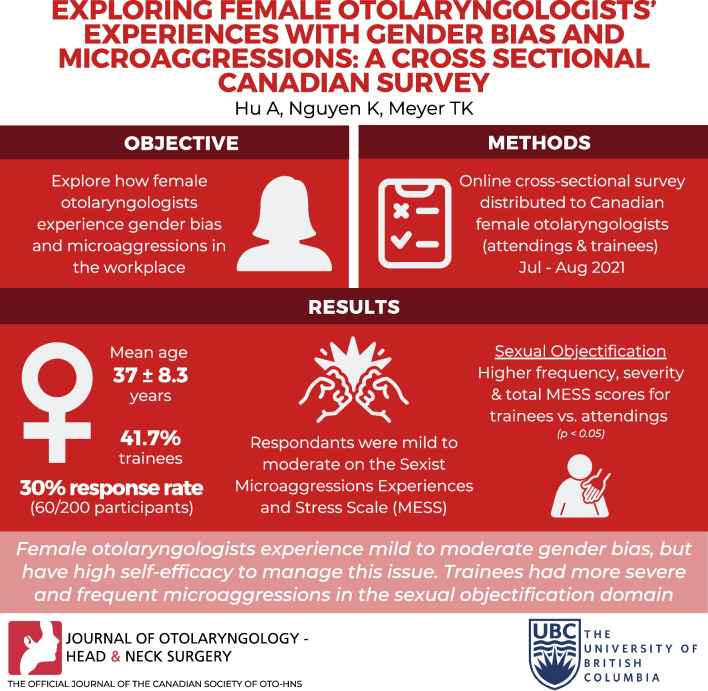

## Background

Gender bias is behavior that shows favoritism toward one gender over another [1]. Gender refers to the socially constructed expectations and roles of women and men [1]. Gender bias may be implicit and affect one’s actions/decision in an unconscious manner [2]. Gender bias can lead to sexism, which is prejudice, stereotyping, or discrimination, on the basis of gender [3].

Studies have identified gender bias as a factor that discourages women from pursuing surgical specialties. Although there are now equal numbers of male and female identifying medical students, fewer women are entering surgical fields, ranging from 14.8% in orthopedics to 40.1% in general surgery [4, 5]. In academic medicine, women comprised only 25% of assistant professors, 17% of associate professors, and 9% of full professors in surgery in 2013 [6]. Gender bias may delay promotions and tenure and discourage rising female leaders [7–10].

Otolaryngology, like most surgical fields in medicine, is traditionally male dominated. The Royal College of Physicians and Surgeons of Canada report that 23.8% of the Canadian Otolaryngology workforce was comprised of women in 2019 [11]. A similar American statistic from the American Association of Medical Colleges reported that 17.1% of practicing American otolaryngologists were women in 2017 [12]. Unfortunately, there are still inequalities for women in otolaryngology. Female otolaryngologists are underrepresented in compensation [13], National Institute of Health funding [14], financial relationships with industry [15], on otolaryngology journal editorial boards [16], and in research productivity [17].

Gender bias has evolved with time. Most scholars concur that overt sexism, like sexual harassment, has become less prevalent since the civil rights movement [18, 19]. More covert, subtle, or “modern” gender discrimination still exists [18, 19]. Originally coined by a Harvard psychiatrist, “microaggressions” are defined as subtle, often unconscious, discriminatory, or insulting actions that communicate demeaning or negative attitudes towards an individual or group [18–20]. Microaggressions have been applied to gender disparity in medicine/surgery [18, 19, 21]. Microaggressions are not benign and have been shown to negatively affect individuals who experience them. Mental health studies have reported increased rates of anxiety, depression, somatic symptoms, decreased well-being, and increased binge drinking among individuals who experience microaggressions [22–26].

The mental health effect of microaggressions on women has been quantified in the literature with the Sexist Microaggressions Experiences and Stress Scale (Sexist MESS) [18]. This validated tool was created as a PhD psychology dissertation. The Sexist MESS correlated significantly with the Mood and Anxiety Symptom Questionaire-Dutch-30 (MASQD30), indicating a positive relationship between sexist microaggressions and general distress, depression, and anxiety. This tool was used in three previous studies exploring microaggressions in female surgeons at a single institution in the United States [19, 27, 28]. In all of these studies, otolaryngologists comprised a very low percentage of the study population—12%, 6%, and 7.7% respectively [19, 27, 28]. Since otolaryngology is a distinct and unique surgical specialty, our goal was to use the Sexist MESS tool to study the effect of gender bias and microaggressions in female otolaryngologists across multiple institutions in Canada.

Self-efficacy (SE) is an optimistic self-belief that one can perform a novel and specific task [29, 30]. Similar to concepts of self-esteem and locus of control, SE describes an individual’s ability to cope with adversity [29, 30]. SE has been shown to be associated with well-being and protective against burnout [31–33]. SE can be quantitated by the validated General Self-Efficacy Scale (GSES) [29].

We hypothesized that: 1. Female otolaryngologists and trainees will score high on Sexist MESS scores. 2. Higher self-efficacy as quantified by the GSES will be associated with lower Sexist MESS scores. 3. Female otolaryngologists and trainees will have high GSES. 4. Trainees will have higher Sexist MESS scores than attendings.

## Methods

This study was approved by the University of British Columbia (UBC) Behavioural Research Ethics Board (REB # H20-04,063). Demographic data, the validated 44-item Sexist Microaggressions Experiences and Stress Scale (Sexist MESS) and the validated 10-item General Self-efficacy scale (GSES) were administered. These tools were adapted from previously published studies that evaluated gender bias in surgery [1, 9, 27, 28].

The Sexist Microaggressions Experiences and Stress Scale (Sexist MESS) was a 44-item validated questionnaire that assessed gender-based microaggressions [18]. This scale explored seven domains: (1) leaving gender at the door (i.e. downplaying of femininity to succeed), (2) sexual objectification, (3) environmental invalidations (i.e. discrimination in the physical environment or systemic policies), (4) invalidation of the reality of women (i.e. denying that gender bias exists), (5) assumptions of traditional gender roles, (6) expectations of physical appearance, and (7) inferiority (compared to men). Each of the 44 items were ranked on a scale from 0 to 3, on two dimensions—a frequency and severity subscore. Previous studies reported that a score of > 2 on any item in the Sexist MESS was considered “commonly occurring” or “moderately bothersome or stressful” [27]. Each dimension subscore ranged from 0 to 132 (i.e. 3 × 44 = 132), with a total Sexist MESS reported as a sum of the frequency and severity subscores, yielding a total score of 0–264 (132 + 132 = 264). A higher score indicated a higher degree of microaggressions, although there was no abnormal cut-off. Since the scale for this outcome measure (out of 264) is a bit awkward and difficult to interpret, our statistician recommended converting it to a scale that is more easily interpreted by the reader. For the purposes of this study, the raw scores were converted to percentages and categories were defined as the following: mild 0–30%, mild to moderate 30–40%, moderate 40–60%, moderate to severe 60–70%, and severe 70–100%.

The General Self-Efficacy Scale (GSES) was a 10-item validated questionnaire that measured self-efficacy [29]. GSES was self-administered and completed in 4 min. Participants rated items on a scale of 1 to 4; scores ranged from 10 to 40. Higher scores indicated higher degrees of SE, although there was no abnormal cut-off. For the purposes of this study, low was defined as 10–20, moderate 20–30, and high 30–40. There are no scores from 0 to 9; thus, our statistician did not recommend displaying the scores as percentages.

Practicing Canadian female otolaryngologists and otolaryngology trainees (fellows and residents) were eligible for this study. For the purposes of this study, a binary definition of gender as male versus female was used; the full spectrum of gender identities could not be explored in the context of this study. Email addresses were gathered from the 17 academic otolaryngology departments and from the Canadian Society of Otolaryngology Women In Otolaryngology group. Exclusion criteria included invalid email addresses and addresses blocked by spam. The survey was administered with the UBC Survey Tool, provided by Qualtrics. This program complies with British Columbia’s Freedom of Information and Protection of Privacy Act (FIPPA) [34]. The survey was distributed by the Dillman’s Tailored Design Method [35]. This methodology was used by the US Census Bureau and Gallup Organization and has been shown to enhance response rates [34]. Participants were contacted four times over an 8-week period (weeks 1, 2, 4 and 7) through email invitations. After week 8, survey enrollment closed. The survey was administered from July to August, 2021. No renumeration was offered for participation. This project was supported by the American Academy of Otolaryngology – Head & Neck Surgery (AAO-HNS), Women in Otolaryngology Endowment Grant, the Canadian Society of Otolaryngology– Head & Neck Surgery, Dr. Elena M. O’Connell Memorial Grant, and the British Columbia Otolaryngology Society Research Grant.

### Statistical analysis

Statistical analysis was conducted by a Masters level statistician with commercial software (SAS 9.4, Cary, NC, USA). Descriptive statistics were used to summarize the characteristics of the study cohort and were displayed as mean (standard deviation) for continuous variables and as counts (%) for categorical variables. Student’s t test was performed to determine if demographic or practice factors were associated with the Sexist MESS. The association between the Sexist MESS and GSES was evaluated with the Spearman correlation coefficient. All p-values were two-sided. P-value < 0.05 was considered statistically significant.

## Results

The study started with 206 eligible email addresses. Five were invalid and one was blocked by spam filter. Of the remaining 200, 60 participants completed the survey, yielding a response rate of 30%. Table 1 shows the demographic data of the study population. Table 2 shows the Sexist MESS scores for the study population. Participants scored mild to moderate on the Sexist MESS – Frequency (mean ± standard deviation) 55.8 ± 24.2 (42.3% ± 18.3%), Severity 46.0 ± 23.9 (34.8% ± 18.1%), Total 104.5 ± 43.7 (39.6% ± 16.6%). Participants scored high on GSES 32.7 ± 5.7 (scores range from 10–40) and there was no difference in GSES scores between attendings 32.1 ± 3.7 and trainees 34.2 ± 3.7 (p = NS). Table 3 lists the five most common and five most bothersome/stressful items in the Sexist MESS. Table 4 shows that the sexist MESS scores were not associated with age, ethnicity, fellowship-training, having children, or years of practice. Analysis of the seven domains of Sexist MESS was conducted with each demographic factor. The only factor that was significant was attending vs trainee, as shown in Table 5. Trainees had higher frequency (p = 0.04), severity (p = 0.02), and total (p = 0.02) MESS scores than attendings in the domain of sexual objectification. Spearman correlation coefficient showed that there was no correlation between GSES and Sexist MESS Frequency (ƿ = − 0.11, 95% CI − 0.37 to 0.17), Severity (ƿ = − 0.17, 95% CI -0.44 to 0.12), and Total Score (ƿ = − 0.09, 95% CI − 0.37 to 0.21).

**Table 1:** Demographic data of the study population (n = 60)

Characteristic	Data	
Age (years) (mean (SD))	37.0	8.3
*Ethnic Background—n (%)*		
Native Person	0	0.0%
Asian	19	31.7%
Black or African	0	0.0%
Hispanic	1	1.7%
White (non-Hispanic)	33	55.0%
Other	7	10.6%
South Asian	1	1.5%
Indian	1	1.5%
Mixed Arabic and White	1	1.5%
Middle Eastern	2	3.0%
Not specified	1	1.5%
*Current job title—n (%)*		
Resident	19	31.7%
Fellow	6	10.0%
Clinical instructor	2	3.3%
Assistant professor	11	18.3%
Associate professor	14	23.3%
Professor	0	0.0%
Community/private practice physician	8	13.3%
*Fellowship trained—n (%)*		
Yes	30	50.0%
No	30	50.0%
*Years in practice (mean (SD))*	9.2	7.4
*Care for children* ≤ *18 years of age—n (%)*		
Yes	30	50.0%
No	30	50.0%
*Hours of work—n (%)*		
Full time (40 or more hours per week)	57	95.0%
Part time (< 40 h per week)	3	5.0%
*Surgery days per week—n (%)*		
0	2	3.3%
1	29	48.3%
2	16	26.7%
3	5	8.3%
4 or more	8	13.3%
*Province of Practice—n (%)*		
Newfoundland	0	0.0%
PEI	0	0.0%
Nova Scotia	3	5.4%
New Brunswick	1	1.8%
Quebec	13	23.2%
Ontario	20	35.7%
Manitoba	4	7.1%
Saskatchewan	1	1.8%
Alberta	4	7.1%
British Columbia	10	17.9%
Data unknown	4	10.0%

**Table 2:** Sexist Microaggressions Experiences and Stress Scale (Sexist MESS) is comprised of a Frequency (0–132) and Severity (0–132) subscore to yield a Total Score (0–264)

Frequency subscore	
Raw score (0–132)	55.8 ± 24.2
Percentage score (%)	42.3 ± 18.3%
Severity subscore	
Raw score (0–132)	46.0 ± 23.9
Percentage score (%)	34.8 ± 18.1%
Total score	
Raw score (0–264)	104.5 ± 43.7
Percentage score (%)	39.6 ± 16.6%

Results are displayed as a raw score and percentage score

**Table 3:** Items with the top five highest frequency and severity scores among the 44 items of theSexist Microaggressions Experiences and Stress Scale (Sexist MESS)

Top 5 Most Frequent Items from Sexist MESS	Domain	Average Score (0–3)
Seen images of female bodies in the media that do not reflect your own body	Environmental Invalidations	2.32
Attempted to “overcompensate” for being female	Leaving gender at the door	1.83
Been told that women have all the same rights as men	Invalidation of the reality of women	1.80
Received unsolicited comments about your physical appearance	Sexual Objectification	1.75
Been asked when you are going to have children before you were asked if you want any children at all	Traditional gender roles	1.73

Top 5 Most Severe Items from Sexist MESS	Category	Average Score (0–3)
Someone has assumed a male was responsible for work you actually did	Inferiority	1.78
A male has ignored or dismissed your contribution at work	Inferiority	1.71
Heard someone in a position of authority say that women are to be blamed when they are sexually assaulted	Invalidation of the reality of women	1.59
Hiding emotions at work in order to not appear too emotional	Leaving gender at the door	1.58
Attempted to “overcompensate” for being female	Leaving gender at the door	1.56

Items are ranked on a scale from 0–3, where a score of > 2 is considered “commonly occurring” or “moderately bothersome or stressful”

**Table 4:** Sexist Microaggressions Experiences and Stress Scale (Sexist MESS) is comprised of a Frequency (0–132) and Severity (0–132) score to yield a Total Score (0–264)

	Sexist Microaggressions Experiences and Stress Scale (Sexist MESS)					
	Frequency (0–132)	P value	Severity (0–132)	P value	Total (0–264)	P value
Factors						
*Age*						
≤ 36 years old	59.6 ± 27.4	0.30	45.6 ± 24.7	0.88	108.0 ± 47.5	0.67
> 36 years old	51.8 ± 21.1		46.6 ± 20.9		102.1 ± 36.6	
*Ethnicity*						
Non-White	49.3 ± 21.7	0.06	41.5 ± 22.7	0.22	93.6 ± 42.4	0.11
White	62.3 ± 25.4		50.2 ± 25.0		114.6 ± 43.8	
*Attending versus trainee*						
Attending	54.1 ± 26.9	0.52	45.9 ± 24.2	0.94	106.0 ± 45.6	0.87
Trainee	58.5 ± 21.1		46.5 ± 24.8		103.9 ± 43.1	
*Fellowship-Training*						
No	59.3 ± 19.8	0.87	47.4 ± 21.2	0.92	106.7 ± 38.8	0.97
Yes	58.2 ± 29.0		48.1 ± 29.3		106.2 ± 55.8	
*Children* ≤ *18 years old*						
No	61.9 ± 23.4	0.12	43.9 ± 24.1	0.51	106.5 ± 43.6	0.97
Yes	51.6 ± 23.6		48.6 ± 24.5		103.5 ± 45.4	
*Years of practice*						
≤ 10 years	62.2 ± 30.7	0.46	54.8 ± 27.6	0.35	117.0 ± 55.8	0.81
> 10 years	54.1 ± 24.3		44.2 ± 27.8		98.3 ± 47.3	

Sexist MESS was not associated with any demographic or practice factor

**Table 5:** Analysis of the seven domains of Sexist Microaggressions Experiences and Stress Scale (Sexist MESS) with the demographic factor of Attending versus Trainee

	Sexist microaggressions experiences and stress scale (Sexist MESS)					
	Frequency	P value	Severity	P value	Total	P value
Domains (no of questions)						
*Leaving gender at the door (4)*						
Attending	6.0 ± 3.2	0.50	5.3 ± 3.4	0.70	11.4 ± 6.2	0.96
Trainee	6.6 ± 3.0		4.9 ± 2.9		11.5 ± 5.6	
*Sexual objectification (8)*						
Attending	7.7 ± 5.2	0.04*	5.3 ± 4.0	0.02*	13.2 ± 7.6	0.02*
Trainee	10.3 ± 4.1		8.2 ± 5.2		18.6 ± 8.1	
*Environmental invalidations (4)*						
Attending	5.9 ± 2.6	0.71	3.6 ± 2.5	0.41	9.6 ± 4.3	0.52
Trainee	6.2 ± 2.7		4.3 ± 3.1		10.5 ± 5.3	
*Invalidation of the reality of women (10)*						
Attending	10.0 ± 6.8	0.17	10.5 ± 7.9	0.34	21.5 ± 13.7	0.33
Trainee	12.6 ± 6.4		12.7 ± 7.5		25.2 ± 13.2	
*Assumptions of traditional gender roles (6)*						
Attending	7.2 ± 5.1	0.22	4.1 ± 4.2	0.97	11.5 ± 8.7	0.53
Trainee	8.8 ± 4.3		4.1 ± 3.8		12.8 ± 6.5	
*Expectations of physical appearance (3)*						
Attending	3.2 ± 2.9	0.24	3.0 ± 2.5	0.73	6.3 ± 4.8	0.37
Trainee	2.4 ± 2.3		2.8 ± 2.9		5.1 ± 5.0	
*Inferiority (9)*						
Attending	12.3 ± 8.1	0.80	12.1 ± 7.6	0.97	25.1 ± 14.8	0.72
Trainee	211.8 ± 6.7		12.2 ± 7.3		23.7 ± 13.6	

Trainees had higher frequency (p=0.04), severity (p=0.02), and total (p=0.02) MESS scores than attendings in the domain of sexual objectification.

*denoted significance, P < 0.05

Each domain has a different potential score based on the number of question items. The frequency and severity are rated on a Likert scale from 0 to 3, and this is multiplied by the number of question items in the domain. For example, inferiority has 9 questions, so it has a possible range from 0 to 27 points for both frequency and severity. The total MESS score is a summation of frequency and severity, so the inferiority domain’s total MESS score ranges from 0 to 54

**Fig. 1:**
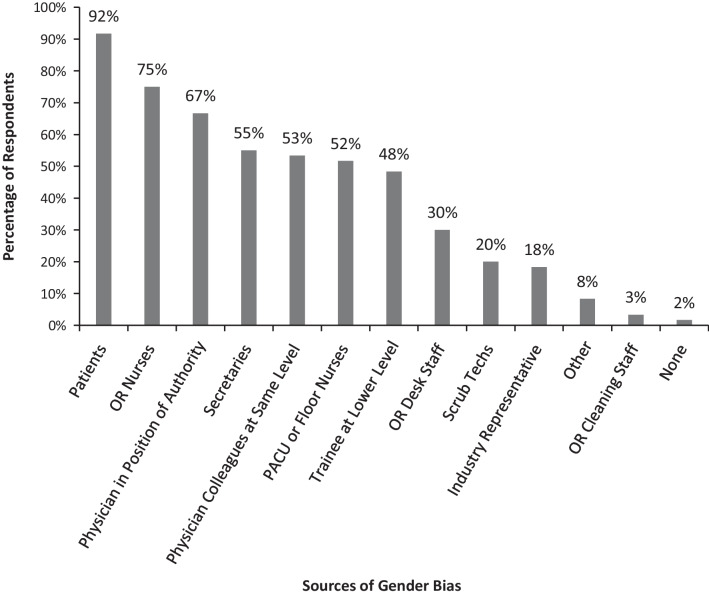
The most common sources of gender bias reported by female otolaryngologists

**Fig. 2:**
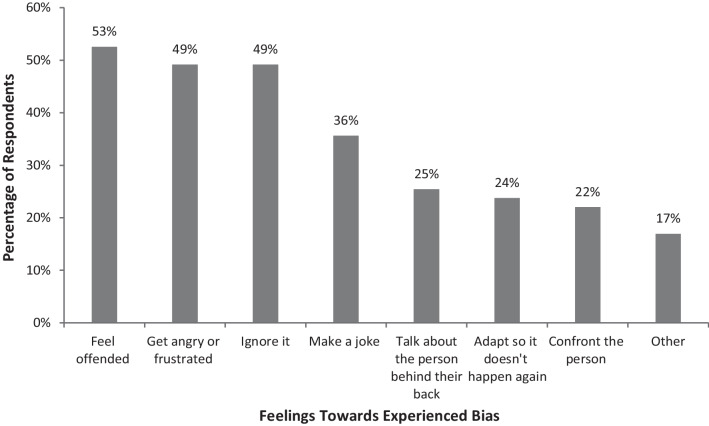
Feelings and responses of female otolaryngologists towards experienced gender bias in the workplace

Figure 1 shows the most common sources of gender bias reported by the participants: 1. patients (92%), 2. Operating room nurses (75%), and 3. Physician in authority position (67%). Figure 2 shows the participants’ feelings and responses towards gender bias: 1. felt offended (53%), 2. ignored it (49%), 3. got frustrated/angry (49%). About 1/3 of participants felt that men were also subject to gender bias. No physical harm or adverse events were encountered, although this topic may have triggered psychological distress among participants.

## Discussion

Three previous single institutional studies used the same outcome measure, the Sexist MESS, to explore gender bias experiences in female surgeons [19, 27, 28]. The first Barnes et al. study of female surgical trainees had 33/50 participants (response rate 66%) [27]. They divided the trainees into female-dominate field (obstetrics/gynecology) and male-dominate fields (all other surgical specialties). Female trainees in male-dominant fields reported more frequent, severe, and stressful microaggression experiences than female trainees in female-dominant fields. Their study had five items in the Sexist MESS that scored over 2, indicating a more severe or significant result, in comparison to our study that only identified one item (Table 2). The second Barens et al. study of female surgeons of all specialties had 65/101 participants (response rate 64%) [19]. The frequency and severity of Sexist MESS scores were higher for trainees than attendings. The variables of non-White ethnicity, having children under 18, and fellowship training were not associated with the Sexist MESS scores. The third Sudol et al. study’s objective was to determine the prevalence of sexist and racial/ethnic microaggressions against female surgeons and anesthesiologists and to assess its’ association with burnout [28]. Using the Sexist MESS, they defined the prevalence of microaggressions as at least 1 mean subscale score for frequency. They also employed the Racial Microaggression Scale and Maslach Burnout Inventory. With a sample size of 652/1609 (response rate 41%) conducted at a single health maintenance organization, they reported that 94% of female respondents reported sexist microaggression and 81% of racial/ethnic minority physicians reported racial/ethnic microaggression. Microaggression was significantly associated with physician burnout, an important mental health and human resource issue in the health care system. This study was able to study the intersectionality of gender and race/ethnicity with microaggression.

This current study reinforces the previous literature that trainees are more at risk for gender bias and microaggressions [27, 36–38]. In the domain analysis of the current study, the frequency severity, and total MESS score was higher for trainees than attendings in the domain of sexual objectification. In the second Barnes et al. study, trainees had higher frequency MESS scores in six of the seven domains and higher severity MESS scores in three of the seven domains [27]. A recent multi-institutional, national survey of American general surgery and surgical specialty residents revealed that the majority (72.2%) of residents had experienced microaggression, most commonly from patients (64.1%) [36]. Only 7% reported these events and nearly one-third (30.8%) of residents experienced retaliation due to reporting of microaggressions [36]. Further studies in plastic surgery and emergency medicine confirmed these results [37, 38]. For example, misidentification as a non-clinician staff, the most common form of microaggression in the emergency medicine study, occurred more commonly with trainees than attendings, more commonly with women than men, and more commonly with non-White than White respondents [38].

In the medical hierarchy, attending physicians may have more authority and power than trainees, who are more vulnerable in experiencing and addressing microaggressions. Attending physicians may be bystanders when these microaggressions occur to trainees. Bystanders are defined as “anyone who becomes aware of and/or witnesses unjust behavior or practices that are worthy of comment or action” [39, 40]. Education and faculty development on how to manage these experiences are crucial and the goal is to support bystanders to become “upstanders”. “Upstanders” are those who take action, intervene, and speak up for trainees/colleagues who are experiencing microaggressions [37, 40]. Universities and teaching faculty members have an ethical and legal obligation to provide a safe learning environment for their trainees. Speaking up for trainees in the face of microaggressions is one aspect of advocating of them.

The current study also investigated the concept of self-efficacy. Self-efficacy has been studied in surgical trainees in relation to well-being and burn out [30–32]. A large cross-sectional survey of all surgical residents at Stanford reported that high self-efficacy was predictive of well-being [30]. Another multicenter study of general surgery residents reported that lower burnout was associated with higher self-efficacy and lower perceived stress scores [31]. A third multicenter study of vascular surgery residents reported that burnout was associated with lower self-efficacy and higher levels of depression [32]. Higher levels of self-efficacy were also found to prevent burnout in other first line responders, like nurses and firefighters [41, 42].

Our study showed that female otolaryngologists have high degrees of self-efficacy, but there was no significant association between GSES and Sexist MESS scores. There may be several explanations for the lack of statistical significance. First, there was a narrow range of GSES scores, so it may be difficult to show a statistical association with another variable. Second, there may be survivorship bias in this study [43]. Female otolaryngologists who survived the rigours of surgical training and stayed practicing in this field were surveyed. Those with lower self-efficacy may have left the field and not been eligible for the study. We do not know the self-efficacy of women who did not complete the study. Lastly, there were no senior full professors who completed the study; albeit, women comprise a very small proportion of full professors in academic medicine [6].

Figure 1 showed that the top source of microaggressions experienced by female otolaryngologists was patients (90%). This was confirmed in three previous studies, which rated patients as the top source of microaggressions experienced by 94% of female surgical residents [27], 80% of all female surgeons [19], and 64.1% of all American surgical residents [36]. Addressing microaggressions that were perpetrated by patients is problematic on multiple levels. Although the conduct of health care providers is directly regulated by industry and hospital standards of professionalism, the conduct of patients is not regulated. Furthermore, patients seek care when they or their loved ones are ill and at their most vulnerable state. Patients and their caregivers often feel they need to advocate for themselves or their loved ones, and they may strike out against health care providers. Health care professionals may feel an ethical obligation to maintain the physician–patient relationship, even in the face of gender bias and microaggressions, and even more egregious behaviour.

Addressing microaggressions and managing gender bias can be challenging. Barriers for physicians to address these issues include fear of retaliation, fear of situational escalation, further discrimination, jeopardizing personal/physical safety, and exclusion by coworkers [44, 45]. Some advocate that the first step to addressing microaggressions is recognizing it [44]. The perpetrator may not be aware of the transgression and may not have had any malicious intent. On a system level, implicit bias and diversity training have been implemented by multiple institutions to reduce these biases. Evidence of successful changes in the workplace, unfortunately, are slow, as it takes a long time to change organizational culture [46, 47].

Figure 2 shows that these microaggressions caused negative feelings and responses in about half of respondents. About half of female otolaryngologists felt offended, got angry, or frustrated. About half of respondents ignored the microaggressions, which is an unhealthy response as it does not address the issue and builds up negative emotions in the victims. Instead, ignoring the microaggressions enables the perpetrator to continue the inappropriate behaviour. Only about one fifth of respondents were empowered to confront the perpetrator. This may educate the perpetrator about the transgression and hopefully break the cycle. Previous qualitative studies have reported that female surgeons have developed resilience—“toughness” or “thick skin” [19, 27]. Some developed coping strategies like using humour; others used increased effort to adapt [19, 27].

One study recommended that future directions should explore how to address these microaggressions in a healthy and productive manner [19]. The Mayo Clinic has developed the GRIT (Gather, Restate, Inquire, Talk It Out) Framework for Addressing Microaggressions: [48] 1. *Gather* your thoughts. Do not overreact with anger. Decide if it is the appropriate juncture to address the perceived microaggression. 2. *Restate* the comment or ask the speaker to restate it. Allow the person to clarify or realize the potential negative impact of the comment. 3. *Inquire*. Dig deeper and seek clarification: Be nonjudgmental. Address the comment rather than making it personal. 4. *Talk* it out. Discuss the potential impact on others and your own perception. This framework aims to promote open, productive communication between all parties, recognizing that we may all be recipients, witnesses, and perpetrators of microaggression.

Gender bias can have potential impact on the hiring process of potential candidates. These unconscious stereotypes have been found to have negative consequences for women who apply for jobs traditionally held by men [49–51]. For example, a systematic review of reference letters for residency and academic medicine faculty positions reported that reference letters for female applicants had more frequent use of doubt raisers and mentions of applicant’s personal life and/or physical appearance [50]. Women were more likely to be described with communal adjectives, like “compassionate”, while men were more likely to be described with agentic adjectives, like “leader”. During interviews, applicants who displayed gender incongruent behaviours (e.g. women who were self-promoting) were rated lower than applicants who behaved in a more gender-congruent manner [51]. Employers are becoming aware of this gender bias and a systematic review discussed interventions to mitigate this [49]. Interventions included providing only job relevant information to raters (e.g. not including parental or marital status), being aware of gender stereotyped behaviour and appearances, and instituting explicit employment equity policies.

We recognize some limitations in this study. Gender is a fluid concept and some otolaryngologists may not fully identify with the binary distinction of cis-man or cis-woman. The authors attempted to build a comprehensive mailing list of all practicing female otolaryngologists by contacting the 17 otolaryngology departments across Canada and the Canadian Society of Otolaryngology Women in Otolaryngology group. Some private practice or community otolaryngologists may have been inadvertently left off the distribution list. Although Table 1 reports that 50.0% (30/60) of the respondents were fellowship trained, the study population included trainees (19 residents and 6 fellows); thus, only 35 respondents were attending surgeons who would have been eligible for completing fellowship training. The proportion of fellowship trained attending surgeons (30/35 = 85.7%) was much higher, so there may have been a higher representation of academic surgeons who have completed a fellowship. Despite employing the Dillman’s Tailored Design Method [34] for survey distribution, the response rate of 30% could be improved upon. The previous studies were all single institution studies [19, 27, 28]. Our sample size of 60 was larger than the first Barnes et al. study (n = 33) and comparable to the second Barnes et al. study (n = 65) [19, 27]. The response rate of 30% is less than the previous two studies’ response rates of 64–66% and the third study’s response rate of 41%, which likely reflects the challenges of a multi-institution study [19, 27, 28]. There may have been a selection bias in which female otolaryngologists who felt strongly about the topic decided to complete the survey. The opinions of those who did not participate were unknown. Since there were no senior full professors who answered this survey, we were unable to comment if more senior female otolaryngologists experienced microaggressions differently than junior female otolaryngologists. About one third of participants felt that men were also subject to gender bias, and this would be an area for future research. Studying the intersectionality of gender with race/ethnicity, religion, and sexual orientation and its effect on microaggression was outside of scope of this current study.

## Conclusions

This was the first multicenter, Canada-wide study exploring how female otolaryngologists experience gender bias and microaggressions in the workplace. These experiences have been shown to have negative effects on individuals who experience them. Validated outcome measures and rigorous survey methodology were used in this study. Female otolaryngologists experience mild to moderate gender bias, but have high self-efficacy to manage this issue. Trainees had more severe and frequent microaggressions than attendings in the sexual objectification domain. Future efforts should help develop skills and strategies for all otolaryngologists to address gender and racial bias experiences, become “up-standers”, and thereby improve the culture of inclusiveness and diversity in our specialty.

## Data Availability

The datasets used and/or analysed during the current study are available from the corresponding author on reasonable request.

## Abbreviations

Sexist MESS: Sexist Microaggressions Experiences and Stress Scale
MASQD30: Mood and Anxiety Symptom Questionaire-Dutch-30
SE: Self-efficacy
UBC: University of British Columbia
REB: Research ethics board
GSES: General Self-efficacy Scale
FIPPA: Freedom of Information and Protection of Privacy Act
AAO-HNS: American Academy of Otolaryngology—Head and Neck Surgery
GRIT: Gather, Restate, Inquire, Talk It Out Framework for Addressing Microaggressions
NS: Not significant
SD: Standard deviation
